# Learning from Reflection on Patient Outcomes Data: How EHR Can Support Trainees in Graduate Medical Education on Inpatient Rotations

**DOI:** 10.5334/pme.1627

**Published:** 2025-05-08

**Authors:** Margaret A. Robinson, Christy Boscardin, Marieke Van der Schaaf, Justin L. Sewell, Glenn Rosenbluth

**Affiliations:** 1 Division of Critical Care Medicine, Department of Pediatrics, University of California San Francisco, San Francisco, California, USA; 2 Departments of Medicine, and Department of Anesthesia, University of California San Francisco, San Francisco, California, USA; 3 Research and Development of Health Professions Education, University Medical Center Utrecht, The Netherlands; 4 Division of Gastroenterology, Department of Medicine, University of California San Francisco, San Francisco, California, USA; 5 Department of Pediatrics, University of California San Francisco in San Francisco, California, USA

## Abstract

**Introduction:**

As healthcare evolves into interdisciplinary, complex, team-based care that often includes shiftwork and sub-specialization, patient outcomes data has become necessary for trainees to engage in reflective practice in clinical environments. However, current practices around collecting and distributing such data to trainees are not effective. Specifically, it is not clear what patient data are significant and compelling to trainees for reflective practice. The goal of our study was to characterize trainee perspectives on what data are meaningful to promote reflective activities for learning in the clinical work environment.

**Methods:**

From 2020–2021, we conducted a longitudinal cross-sectional study to assess trainee interest in clinical outcomes data. Over 14 days, pediatrics and internal medicine residents doing inpatient work at the University of California San Francisco completed surveys corresponding to recently opened patient charts.

**Results:**

958 surveys were completed by 41 participants (average 23 unique patient encounters per participant). Trainees expressed interest in follow-up for 32.9% of encounters (n = 315/958), most often to ‘learn if something significant or unexpected happened.’ Trainees most often desired follow-up patient data when they had made significant decisions or felt responsible.

**Discussion:**

Trainees were interested in clinical outcomes data for a limited number of patient encounters, highlighting challenges with current strategies to promote reflective practice using clinical outcomes data. While refinement of such approaches continues through consideration of what trainees find meaningful in data, understanding motivating and demotivating factors in trainees’ outcomes data-seeking behaviors will also be crucial for success in using such data for learning opportunities.

## Introduction

Graduate medical education (GME) trainees work and learn in increasingly complex clinical environments that function through team-oriented, interdisciplinary approaches often relying on shiftwork and sub-specialization to effectively implement patient care. Essential to learning at work is the ability for a trainee to reflect on their experience, a deliberate process of thinking about and interpreting situations or events for better comprehension and for learning [1,2]. See *box A* for a descriptive vignette of how a trainee might practice reflective activities for learning on a complex inpatient rotation.

**Box A Vignette of a trainee on an inpatient rotation**

Imagine intern Simon on a busy hospital medicine rotation. Between listening to his patients, examining them, placing orders for them, presenting them, participating in diagnostic and therapeutic reasoning, and discussing their cases with consultants, there is hardly time for bio breaks, much less reflection on learning. He cares for a variety of patients, ranging from one admitted with shortness of breath and no underlying medical conditions, to another requiring a monoclonal antibody infusion for their rare autoimmune disease, to another needing inducement and characterization of their seizures for surgical planning. He finishes his rotation the day after admitting the healthy 30-year-old with shortness of breath and ground glass opacities on chest imaging who was undergoing further work-up. Simon had initiated treatment for tuberculosis and found himself looking up the patient’s course weeks later, surprised to find that the infiltrates were leukemic in origin. He never does look up the patient who needed the antibody infusion so does not learn that the patient developed a central line associated bacterial infection (CLABSI) nor the patient with epilepsy who had their seizures mapped and the seizure focus successfully resected by the neurosurgical team. What motivates Simon to look up one patient versus another? How does he decide where the most meaningful learning upon reflection may occur and how does he enact this learning?

Learning from reflection is a critical skill for graduate medical education learners and practicing providers alike. It is labelled in a variety of ways including quality metrics, assessment, or practice-based learning and improvement [3,4]. Reflective activities allow a trainee to evaluate both their own role in a patient’s clinical care as well as the performance of the entire team. Their assessment then leads to a wide range of learning opportunities for the trainee, from refinement or expansion of differential diagnoses and efficacy of treatments to identification of healthcare system failures that contribute to poor patient experiences. Such reflective practices exist within a mindset universally accepted as fundamental not only to physician development but as a career-long practice: one of curiosity and life-long learning [5,6]. One element necessary for reflection to occur is the provision of clinical outcomes data that provides critical information for reflection on patient care and decision-making. Broadly speaking, the National Institute of Health (NIH) defines clinical outcomes as “a result that describes or reflects how an individual feels, functions or survives”. The NIH further specifies four types of outcomes data: patient-reported, observer-reported, clinician-reported, and performance [7]. The representation of such outcomes in EHR data ranges from unexpected test results or transfer to a higher level of care to aggregated quality metrics such as re-admission rates. Indeed, many professional societies mandate that practicing providers review patient outcomes for maintenance of certification, and governing GME associations have also recognized the importance of such data and implemented requirements for individualized provision of patient outcomes data for performance-based feedback.

Despite these mandates, current practices around collecting and distributing such data to trainees (e.g., via dashboards or required curricula for self-reflective practice) have not been proven effective in enhancing learning [8,9]. Part of the challenge lies in the logistics of how trainees obtain these outcomes data and also in the facilitation of the trainee’s cognitive process of meaning-making necessary for learning to occur. First, in addressing logistical issues, electronic health records (EHRs) present an opportunity to provide such patient outcomes data to trainees. At an individual level, the most basic EHR-based methods for follow-up include keeping lists of patients for whom there is a desire to check on at a future date (i.e., “chart-stalking”) [10]. Even though this technique is seemingly simple, resident self-directed follow-up on patients occurs infrequently – less than 40% of the time in one inpatient study [11]. At a programmatic level, building algorithms to automate list-making through patient attribution has afforded a more efficient opportunity for clinical follow-up [12]. GME training programs are attempting these large-scale interventions and often struggle to successfully deliver clinical outcomes information [13–15]. Specifically, current automation approaches have proven challenging due to lack of concordance between the patient lists auto-generated from the attribution algorithm and user-generated patient lists. This is due in part to the fact that residents who care for the same patient are often labelled in the system as a generic team member, if identified at all, unlike the faculty who will be entered as the attending of record [16]. Thus, there is a lack of ability to attribute patient-specific information to residents in the same manner that attendings are attributed. This discrepancy in attribution between resident and attending highlights the fact that trainees are not automatically granted “ownership” over patient care, and they are far more likely than attending physicians to regularly move into and out of healthcare systems based on their rotation schedules. This discordance in attribution of patients may contribute to lack of interest or meaningfulness to individual trainees’ perception of the patient outcomes data.

The challenges around the residents’ cognitive process of meaning-making of clinical information are less well known. The complex learning environment, with shift work and sub-specialization, likely contributes to trainees’ motivation and which experiences they select for reflective practice. Previous work has shown that trainees and supervisors apply different criteria to identify populations of patients for whom they want data on clinical outcomes, suggesting there may be nuance in the meaning-making for trainees [13]. Further investigation of trainee engagement with clinical outcomes data, including assessment of the types of data desired and what trainees consider meaningful outcomes, is necessary to better facilitate development of reflective practices and ultimately learning opportunities in the complex clinical learning environment. Addressing this gap could benefit post-graduate programs with the development of clinical outcome tools, trainees with optimizing opportunities for learning, and ultimately patients for the improvement of clinical care. In this longitudinal study using real-time EHR data, we sought to better understand trainees’ perspectives on attribution and outcomes data with our research question: what type, why and on which patients do trainees want clinical follow-up during their clinical rotations?

## Methods

### Study Design

We conducted a longitudinal, cross-sectional survey study. Participants scheduled for inpatient rotations in the Departments of Internal Medicine and Pediatrics at the University of California San Francisco were recruited via email. We explicitly chose team-based, inpatient rotations given the challenges related to attribution (as opposed to patient encounters where only one trainee is involved such as the emergency department or outpatient clinic). Residents and fellows who responded with interest received an explanatory video and a link to an online baseline demographic questionnaire and consent. Participants then received online surveys asking about their role in the care of specific patients whose charts they had recently opened and querying their interest in follow-up on those specific patients’ outcomes. Referencing specific patients helped participants to express their preferences as they pertained to actual patients and desired follow-up, rather than in generalities or hypotheticals only. Surveys were sent every other day for two weeks, for a total of seven survey-days, beginning on the second day of the rotation. On each of the seven survey-days, trainees were sent an email containing surveys for up to 10 unique patients whose charts they had opened in the prior 24 hours; the maximum number of surveys sent per trainee was 70 total. Participants received a $100 gift card if they completed at least 70% of the surveys. The institutional ethical review board at University of California San Francisco approved this study (RB Number 19–28700).

### Participants

Inclusion criteria were residents and fellows across the internal medicine residency program, pediatrics residency program, adult critical care fellowship program, and pediatric critical care fellowship program at a single institution who were rotating on hospital medicine and intensive care unit (ICU) services from winter 2020 to spring 2021. Other categorical residents were included if rotating through one of these hospital medicine or ICU rotations. These specialties were chosen given their consistent involvement in hospital-based rotations where it is common for trainees to assume a ‘primary provider’ role. We felt that this relationship would help focus their follow-up on actual patients, as prior research found that residents needed to feel that they contributed to patient care to want follow-up [13]. While there are differences in the responsibilities of residents and fellows, they are part of the same care team, connected by shared interactions and context, and therefore we included both populations in the analysis. We included the question of trainee role in the surveys to be able to select those who viewed themselves as a ‘primary provider.’ For those meeting inclusion criteria, there were no specific exclusion criteria. We emailed an introduction to our study to 223 trainees scheduled for hospital medicine or ICU rotations during the study period. Through this convenience sampling, 42 (n = 42/223, 18.8%) indicated interest and consented to the study. Thirty-six trainees (n = 36/42, 85.7%) completed at least 70% of their surveys.

### Survey

The baseline questionnaire included questions about trainee demographics including year of training and upcoming inpatient rotation start date. The patient-level clinical follow-up surveys included five multiple choice items eliciting the following: 1) what role the provider played in the past 24 hours, 2) why the provider opened the chart, 3) whether they were interested in clinical follow-up, 4) what type of clinical follow-up they were interested in, and 5) why they were interested in follow-up (Appendix A). For questions 4 and 5, the participant could select all answers that apply. Surveys were piloted with four residents including cognitive interviews which informed modifications and clarifications to survey questions. Types of clinical follow-up (i.e., feedback from a supervisor or colleague about clinical management, follow-up if something significant or unexpected occurred, or inclusion in a report with personalized aggregated quality metrics) were chosen based on these interviews as well as the Accreditation Council for Graduate Medical Education program requirements [14]. Content validity was attained by selecting survey items (i.e. choices for ‘reasons why trainees were interested in follow-up’) based on prior work that included expert opinion and prior literature that were then further informed by the cognitive exit interviews [13,17].

### Data Analysis

Responses were summarized using descriptive statistics including means, standard deviations, and frequencies. We used chi-square tests to compare responses from the cases indicating desire for clinical follow-up vs. not. If a trainee was surveyed about the same patient multiple times (i.e., on subsequent days), only the first survey response for the unique patient was included in the analysis to eliminate duplication. To account for nested structure of the data (multiple surveys completed by trainees), we conducted mixed effects logistic regression analysis. We used a mixed-effects logistic regression model to examine the association between demographic variables and the binary survey outcome (desire for clinical follow-up vs. not). Given that each participant provided multiple responses, we included a random intercept for each participant to account for within-subject correlation. Fixed effects included key demographic predictors (e.g., primary provider, specialty, etc.), while the participant-level random intercept controlled for individual differences in baseline response tendencies. Model estimates were reported as odds ratios (ORs) with 95% confidence intervals (CIs) to quantify the strength of associations. Statistical significance was set at p < 0.05. All analyses were completed using the statistical package for social sciences (SPSS) version 28 (SPSS Inc., Chicago, Illinois).

## Results

From 2020–2021, 958 total patient-provider surveys were included for descriptive analysis, an average of 23 surveys per trainee enrolled (range 5–61 surveys). Characteristics of trainees are in Appendix B. Overall, trainees expressed a desire for follow-up information in 32.9% of encounters (n = 315/958, Table 1). On average, an individual trainee was interested in follow-up on 43% (SD 13%) of their own patient encounters.

**Table 1:** **Interest in follow-up based on role**. *Interest in clinical follow-up by resident/fellow in patient encounter, including by indicated role in patient care*. * P-values are based on mixed effects model.


	DYAD/PAIRS OF PROVIDER AND FIRST TIME OPENED PATIENT FILE ARE YOU INTERESTED IN FEEDBACK/FOLLOW-UP ON THIS PATIENT?		ODDS RATIO	CI	P-VALUE*

	NO (N = 643)	YES (N = 315)			

What was your role in this patient’s care during the past 24 hours: (N %)					

*I was **primary** for this patient*	279 (51.4%)	264 (48.6%)	10.37	6.55–16.41	0.000

*I was in a **cross-covering** role*	112 (80.0%)	28 (20.0%)	2.74	1.51–4.97	0.018

***Other** role*	252 (91.6%)	23 (8.4%)	—		


There was no significant difference between pediatrics and internal medicine residents (28.9% (n = 117/405) vs. 33.4% (n = 137/410), p = 0.56, OR = 0.81, CI = 0.60–1.09) in the proportion who desired clinical follow-up (Table 2). When compared by post-graduating training year (PGY), PGY-1 trainees desired follow-up 37.5% (n = 117/312) of the time, PGY-2 trainees desired follow-up 22.9% of the time (n = 65/284, p = 0.001, OR = 0.50, CI = 0.35–0.71), PGY-3 trainees 33.8% of the time (n = 74/219, p = 0.38, OR = 0.85, CI = 0.59–1.22), and PGY-4 through PGY-7 trainees 41% of the time (n = 59/143, p = 0.61, OR = 1.17, CI = 0.78–1.75) indicating differences across the trainee levels. Participants were more interested in follow-up on encounters with higher severity of illness (SOI) scores (35%, n = 224/646) than encounters with lower SOI scores (27%, n = 54/201) which was statistically significant (p = 0.04, OR = 0.69, CI = 0.49–0.98). When comparing the desire for follow-up data based on the provider roles, trainees identified as the primary provider on 543 encounters; they wanted follow-up on 48.6% of the cases (n = 264/543) compared to 20% (n = 28/140) for cross-covering role and only 8% for those in other roles (n = 23/275) (Table 1).

**Table 2:** **Interest in follow-up based on specialty, SOI score and PGY**. Interest in clinical follow-up on patient encounter, including by residency program, patient severity of illness and year of post-graduate training. *P-values are based on mixed effects model.


	DYAD/PAIRS OF PROVIDER AND FIRST TIME OPENED PATIENT FILE ARE YOU INTERESTED IN FEEDBACK/FOLLOW-UP ON THIS PATIENT?		ODDS RATIO	CI	P-VALUE*

	NO (N = 643)	YES (N = 315)			

**Specialty (N %)**					

Pediatrics Residency	288 (71.1%)	117 (28.9%)	0.81	0.60–1.09	0.564

Internal Medicine Residency	273 (66.6%)	137 (33.4%)	—		

**Patient Severity of Illness (N %)**					

SOI Low (1 or 2)	147 (73.1%)	54 (26.9%)	0.69	0.49–0.98	0.040

SOI High (3 or 4)	422 (65.3%)	224 (34.7%)	—		

**What is your position? (N %)**					

1st-year resident	195 (62.5%)	117 (37.5%)	—		

2nd-year resident	219 (77.1%)	65 (22.9%)	0.50	0.35–0.71	0.001

3rd-year resident	145 (66.2%)	74 (33.8%)	0.85	0.59–1.22	0.381

Fellow	84 (58.7%)	59 (41.3%)	1.17	0.78–1.75	0.614


### Characteristics of Desired Follow-up

**Figure 1:**
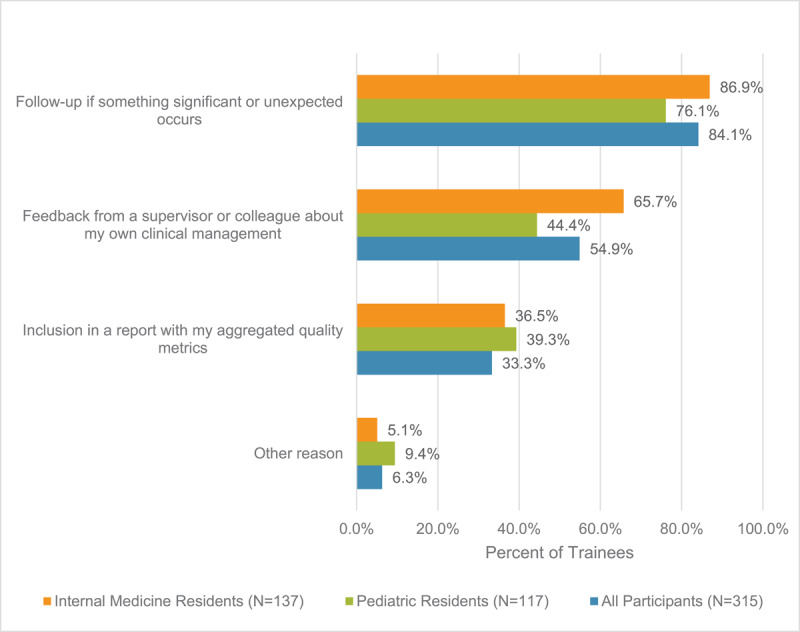
**Types of Follow-up Desired by Trainees**. Types of follow-up that trainee was interested in as form of follow-up. Participants able to select multiple responses.

The most desired type of follow-up information was: ‘[to be notified] if something significant or unexpected happened,’ followed by ‘feedback from a supervisor or colleague on the trainee’s clinical management’. The least desired type of follow-up information was information related to aggregated quality metrics. These findings were consistent across pediatrics and internal medicine trainees. See Figure 1.

**Figure 2:**
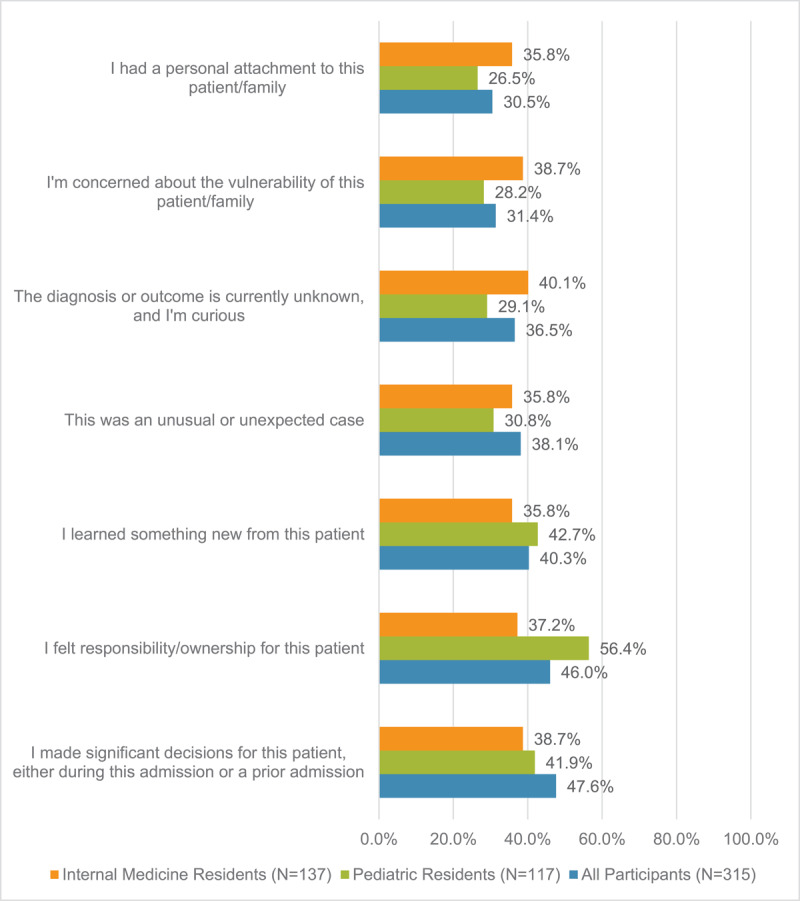
**Reasons indicated for trainee interest in follow-up**. Reasons for trainee interest in clinical follow-up. Participants able to select multiple responses.

From most to least cited, the motivations for wanting outcomes data were if the trainee made significant decisions for the patient (n = 150/315, 47.6%), if the trainee felt responsibility and ownership for the patient (n = 145/315, 46.0%), if the trainee learned something new from the patient (n = 127/315, 40.3%), an unusual or unexpected case (n = 120/315, 38.1%), the diagnosis or outcome was currently unknown and the trainee was curious (n = 115/315, 36.5%), the trainee was concerned about vulnerability of the patient/family (n = 99/315, 31.4%) and if the trainee had personal attachment to the patient/family (n = 96/315, 30.5%) (Figure 2). The reasons for wanting follow-up varied slightly by specialty.

## Discussion

In this study, we explored GME trainees’ interest in receiving outcomes data to reflect on clinical encounters and the nature of that attribution and data. Trainees desired outcomes data on 32.9% of their inpatient clinical encounters; this was higher at 48.6% if participants viewed themselves as primary provider but still reflects less than half of their clinical encounters on inpatient rotations. We identified 543 instances of trainee attribution as a primary provider role to a specific patient. When trainees did want outcomes data, most often it was to be notified if something significant or unexpected happened after their care for the patient ended. Leading contributing reasons for wanting outcomes data were if the trainee made a significant clinical decision or if they felt responsibility or ownership.

Our study highlights challenges associated with conceptualizing, designing, and implementing strategies to promote reflective practices on patient encounters for learning in the clinical workplace, including the analysis of a broad spectrum of performance to guide trainee learning from practice and potential improvement for patient care [18]. Trainees’ limited interest in follow-up in our study suggests that there may be a misalignment between governing bodies’ expectations to provide individualized patient outcomes data and trainees’ lived workplace learning experiences. Often when designing EHR-based data platforms, the thought has been if the EHR could correctly identify a trainee as a primary provider for a patient (via algorithm based on note author, digital footprint, etc.), then that would be an encounter for which the trainee would desire follow-up (i.e., “if you build it, they will come”) [19]. Based on this assumption, efforts have focused on validating attribution algorithms to correctly identify a primary provider, including those who are less visible in the chart (i.e., senior residents, fellows) [16]. However, our findings show that even when trainees identify as primary provider, they often are not interested in knowing the clinical outcome.

A different perspective may consider whether institutions are providing appropriate patient assignments to trainees. Our results correspond with previous work that investigated what constitutes a meaningful encounter for review. Trainees were not interested in receiving clinical follow-up data on patients broadly (contradicting the views of attendings) and rather on patients for whom they felt they contributed to the decision-making or had clinical ownership [13]. There seems to be important nuance in the types of patients on whom trainees would like outcomes data, which goes beyond EHR-based factors such as being a primary provider. We hypothesize that other factors likely contribute. For example, some trainees may have little intellectual curiosity regarding patients who require either highly specialized or algorithm-based care, as the trainee may feel little agency or ownership in such cases. Programs could consider addressing these issues through technical, organizational or cultural changes. Technically, future attempts to use EHR for meaningful and acceptable follow-up may need to consider a more personalized attribution system which goes beyond algorithms that accurately identify primary providers. Thus, a report could be narrowed to a smaller selection of a trainee’s patient list from which the trainee would find the outcomes more consistently meaningful. Programs could also reorganize team structures to better align trainees with patients whom they perceive their care of and resulting outcomes data may produce learning opportunities. Finally, programs could consider cultural shifts that would more often include trainees in decision-making or other efforts to promote patient ownership [20].

Alternatively, there may be a need to shift trainee attitudes toward patient encounters. One study showed that trainees can view exercises in practice-based learning and improvement as a service to the institution rather than learning opportunities [21]. Further work could investigate strategies to help residents “find the learning” in encounters that they don’t find meaningful. There is no consensus on the number of cases a trainee should experience to achieve competency. While trainees may not find multiple admissions for a common chief complaint as compelling as rare conditions or unusual presentations, their participation in each and every case can be used to further develop and refine illness scripts. In addition, there is a service component to the trainee’s role – in other words, sometimes the system and patients rely upon trainees for work defined as more administrative and trainees historically view this aspect of their role as not one for learning [22]. Exploring this attitude may provide more information on the developmental stage of a trainee in comparison to an attending.

Our findings also underscore the importance of choosing meaningful outcomes to follow. As suggested by our results, focusing on the ‘unexpected’ would be meaningful for trainees. Such a focus has also shown to be important for patient care: the unexpected event of ‘change in diagnosis’ is associated with increased patient mortality [23]. In the EHR, these events could be linked to documentation around a clinical deterioration requiring escalation of care or a readmission (i.e. an ICU transfer or code event note). Future possibilities exist for finding such events with the evolution of artificial intelligence in EHR. Learning from such events then promotes ongoing improvement in patient care (i.e., refining an illness script for future diagnoses). Furthermore, focusing on surprising outcomes highlights the importance of learning as a process of ongoing participation rather than of the static acquisition of skills and knowledge – in other words, it reinforces the habits of a life-long learner [24].

Our study further suggests the clinical learning environment may afford only limited opportunities for trainees to feel they have made significant decisions or are primarily responsible for patient care – the most common reasons for wanting outcomes data in our study. Others have noted that time constraints limit opportunities for trainees to explore and reflect on learning outcomes [25]. Another possible explanation for lack of trainee interest in outcomes data may have been stress or work pressure and, possibly, burnout. Certainly, burnout can lead to less engagement, and, when unengaged, trainees are less likely to want to seek follow-up and improve [26,27]. Research has shown that burnout is highest mid-training, and our study followed this same pattern with PGY-2 trainees being the least interested in receiving follow-up on the care of their patients [28]. Our work supports ongoing concerns about the detrimental effects of burnout during training including inability to be motivated to learn from clinical care and follow-up.

Ultimately, motivation appears to be a core feature of desire (or lack thereof) for follow-up, and targeting ways to encourage motivation in the learning environment may cultivate more interest in follow-up. For example, a simple solution would be to give more autonomy to augment trainee motivation [29]. However, this may not always be feasible in the complex care that often characterizes inpatient medicine; more creative solutions could include giving autonomy in other forms, such as allowing trainees to choose which patients they would receive follow-up on as they care for them in real-time. Such autonomy may more effectively promote motivation for trainees to capitalize on performance-based learning opportunities. Our study only explored possible motivations for wanting outcomes data, but demotivating factors should also be considered such as time constraints or the sheer amount of data available in EHR. At minimum, further work to understand trainees’ motivation or demotivation for follow-up on their patients may identify areas for educators and supervisors to focus in order to foster trainees’ perceptions of patient care ownership and motivation for patient follow-up. Without this understanding, attempts at harnessing ‘big data’ for medical education may never reach their full potential.

There were several limitations to our study. It was conducted at a single site and results may reflect a culture unique to the institution. Data collection occurred in the height of the COVID-19 pandemic, which may have created a different patient census than was typical for trainees and more extraneous load affecting trainee motivation. The use of convenience sampling may have introduced selection bias in favor of trainees with interest in the topic and bandwidth to participate. Given the exploratory nature of our study, we felt this was reasonable. Furthermore, if such selection bias is present and our study participants were those with greater interest in following up, this would imply even greater apathy toward follow-up among GME trainees in general, further underscoring the importance of addressing this phenomenon. Our work also focused on two specialties and was restricted to rotations where the trainees were on inpatient wards teams and may not be generalizable across specialties or clinical settings. Finally, survey burden may have contributed to less thoughtful or accurate responses from trainees.

EHRs hold a wealth of data that have not yet been fully utilized to provide follow-up to trainees. Our work identifies elements of the types of information that trainees would be interested in which is crucial to maximizing the wealth of information available from the EHR. Our work also revealed potential challenges related to trainee motivation to investigate outcomes data and may represent an area of research that could improve reflective activities in GME. Ultimately, EHR outcomes data may offer a way to close the feedback loop that is the final process in reflective learning.

## Additional File

The additional file for this article can be found as follows:

10.5334/pme.1627.s1 Supplemental Content. Appendix A–Appendix B.

## Author note

This manuscript has not been previously published and is not under consideration in the same or substantially similar form in any other journal.

All individuals listed as authors are qualified for authorship and all who are qualified to be authors are listed as authors on the byline.
