# Supplementary material for: Learning from Reflection on Patient Outcomes Data: How EHR Can Support Trainees in Graduate Medical Education on Inpatient Rotations
Source: Perspect Med Educ. 2025 May 8;14(1):264–73. doi: 10.5334/pme.1627 (PMC12063598; doi:10.5334/pme.1627)
Supplement: Supplemental Content. — Appendix A–Appendix B. [file pme-14-1-1627-s1.pdf]

Supplemental Content

Appendix A. Participant survey

Survey for Residents and Fellows for Patient-Provider Encounter

1. Which of these best describes your role in this patient's care during the past 24 hours?
   - ☐ I was in a primary role, either as intern, senior resident, or fellow
   - ☐ I was in a cross-covering role (e.g. at night, during a clinic-afternoon, etc.)
   - ☐ I was not directly responsible but this patient was on my team
   - ☐ The patient was not on my team nor was I providing direct care (e.g. potential admission that didn't go to my team)
   - ☐ I was in a consultative role (e.g. RRTs, ED evaluation)

2. Why did you open this patient's chart? (choose the best descriptor)
   - ☐ I provided direct patient care during the past 24 hours
   - ☐ I opened the chart to complete a task for a colleague or another administrative reason
   - ☐ I was curious/interested in the case
   - ☐ I opened the chart in error
   - ☐ I opened the chart for another reason: ___________ [please insert]

3. Are you interested in feedback/follow-up on this patient?
   - ☐ Yes
   - ☐ No

4. What type of feedback/follow-up would you be interested in? (check all that apply):
   - ☐ Feedback from a supervisor or colleague about my own clinical management
   - ☐ Follow-up if something significant or unexpected occurs (e.g. unexpected test result, transfer to different level of care, code)
   - ☐ Inclusion in a report with my aggregated quality metrics (e.g. my own readmission rate, my immunization rate, time to antibiotics, etc.)
   - ☐ Other: ______________ [please insert]

5. What factors contribute to your interest in feedback or follow-up?: (check all that apply) (skip logic if Not Interested)
   - ☐ The diagnosis or outcome is currently unknown, and I'm curious
   - ☐ This was an unusual or unexpected case

- ☐ I made significant decisions for this patient, either during this admission or a prior admission
- ☐ I learned something new from this patient
- ☐ I had a personal attachment to this patient/family
- ☐ I'm concerned about the vulnerability of this patient/family
- ☐ I felt responsibility/ownership for this patient
- ☐ Other: _______________ [please insert]

Appendix B. Characteristics of Participants. *Characteristics of participants including training program and post-graduate training year.*

Pediatrics (n=14)	
PGY-1	6
PGY-2	4
PGY-3	4
Female	11
Internal Medicine (n=22)	
PGY-1	13
PGY-2	6
PGY-3	3
Female	10
Pediatric Critical Care Fellowship (n=4)	
1st Year	1
2nd year	1
3rd year	1
4th year	1
Female	3
Adult Critical Care Fellowship (n=1)	
1st Year	1
Female	0
